# Design of Sensor Data Processing Steps in an Air Pollution Monitoring System

**DOI:** 10.3390/s111211235

**Published:** 2011-11-28

**Authors:** Young Jin Jung, Yang Koo Lee, Dong Gyu Lee, Yongmi Lee, Silvia Nittel, Kate Beard, Kwang Woo Nam, Keun Ho Ryu

**Affiliations:** 1 Korea Institute of Science Technology and Information, 245 Daehangno, Yuseong, Daejeon 305-806, Korea; E-Mail: yjjung@kisti.re.kr; 2 IT Convergence Technology Research Laboratory, Electronics & Telecommunication Research Institute, Daejeon 305-700, Korea; E-Mail: yk_lee@etri.re.kr; 3 Database/Bioinformatics Lab, Chungbuk National University, Cheongju 361-763, Korea; E-Mails: dglee@dblab.chungbuk.ac.kr (D.G.L.); khryu@dblab.chungbuk.ac.kr (K.H.R.); 4 School of Computing and Information Science, University of Maine, Orono, 5711 Boardman Hall, Rm. 344, Orono, ME 04467, USA; E-Mails: nittel@spatial.maine.edu (S.N.); beard@spatial.maine.edu (K.B.); 5 Department of Computer and Information Engineering, Kunsan National University, Kunsan 573-701, Korea

**Keywords:** environmental monitoring system, geosensor network, sensor data processing steps, context aware model, air pollution monitoring

## Abstract

Environmental monitoring is required to understand the effects of various kinds of phenomena such as a flood, a typhoon, or a forest fire. To detect the environmental conditions in remote places, monitoring applications employ the sensor networks to detect conditions, context models to understand phenomena, and computing technology to process the large volumes of data. In this paper, we present an air pollution monitoring system to provide alarm messages about potentially dangerous areas with sensor data analysis. We design the data analysis steps to understand the detected air pollution regions and levels. The analyzed data is used to track the pollution and to give an alarm. This implemented monitoring system is used to mitigate the damages caused by air pollution.

## 1. Introduction

Sensor networks are new instruments useful to detect the conditions in remote places in the physical world [1] in environmental monitoring applications such as pollution monitoring, transportation management, and intrusion detection. Sensor networks are especially effective in the applications that involve checking a large geographic region or under dangerous conditions, because people can’t check the condition of those areas all the time. There are various kinds of examples of their use, such as analyzing dramatic climatic changes, monitoring red tide outbreaks in the ocean, and tracking rare animals in a jungle. Environmental monitoring applications need sensors, communication, and computer technologies with knowledge about the environment [2]. For example, the key characteristics of EOFS (Environmental Observation and Forecasting Systems) are centralized processing, high data volume, QoS (Quality of Service) sensitivity, backwards data flow, extensibility, and autonomous operation [3].

Pollution can causes serious health consequence and negative social and economic damage. Thus, appropriate preparation against pollution emergencies is strongly required. The observed data processing steps in a monitoring system are required to understand and cope with the detected air pollution.

In this paper, we present an air pollution monitoring system designed to give an alarm about a potentially dangerous area. First, we design the data analysis steps to understand the status of air pollution at a remote location. These steps include the raw sensor data abstraction and the region extraction with dangerous conditions by using the context model [4]. This context model is used in our system as a “mold” for data. When data is imported into the model, a data summary is created as a formal presentation of the model. In our system, the steps derive the higher-level and interpreted qualitative information such as current and potential pollution areas depending on the progress of pollution. To reduce severe damage and recovery costs, our system provides an alarm and safety guidelines for these areas.

## 2. Related Work

To detect phenomena in the physical world, environmental monitoring applications are being actively researched according to the advances in wireless sensor networks and observation technologies [5,6]. In some applications for centralized data analysis, sensor networks are designed to transmit the observed data to some central processing site(s). They combine various kinds of sensor data with geospatial information to understand the changes of environmental conditions in numerous monitoring applications such as CORIE [3], PODS [7], GLACSWEB [8], SIFTS [9], PORTS [10], CMOP [11].

In order to guide vessel transportation and forecasting, CORIE uses sensor stations in the Columbia River to get various environmental data [3]. In a centralized computer farm, 2-D and 3-D fine-grain environmental models are derived from the detected environmental conditions such as temperature, salinity, water levels, and flow velocities. The results of the models have been utilized for online control of vessels, marine search and rescue, and ecosystem research and management [3]. The PODS project monitors the rare and endangered species of plants with high-resolution cameras, temperature, and solar radiation [7]. The GLACSWEB project monitors the behavior of ice caps and glaciers for understanding the Earth’s climate [8]. SIFT (Short-term Inundation Forecasting for Tsunamis) is a tsunami forecasting system used in NOAA tsunami warning centers. SIFT detects a tsunami and gives a warning to coastal communities of the impending danger [9]. Ocean floor bottom pressure recorders (BPRs) are used for detecting sudden water pressure changes and transmit the measured value to a moored buoy on the ocean surface. It checks the conditions of ocean such as tsunami amplitudes, flow velocities, and arrival times for offshore, coastal, and inundation areas. To provide useful information, SIFT also employs Java/Jini technology for utilizing data assimilation and inversion schemes with Fortran-based numerical models [9]. PORTS (Physical Oceanographic Real-Time System) is a decision support tool to promote navigation safety, improve economic efficiency, and protect coastal resources [10]. It integrates real-time environmental observations, forecasts and other geospatial information to support safe and cost-efficient navigation like a vessel traffic system for waterways by measuring and predicting water levels, currents, salinity, and meteorological parameters such as winds, atmospheric pressure, air and water temperatures [10]. CMOP (Center for Coastal Margin Observation & Prediction) shows the challenges for coastal-margin science by considering modeling, simulation, sensors, and platforms [11]. The goal of the Coastal Margin Observatories is to describe and understand the river-to-ocean environments by integrating modeling systems, heterogeneous observation networks, and information delivery systems [11]. FloodNet, which is an example of pervasive computing, tries to check a functional floodplain condition at a particular location by using wireless sensor network with automated adaptive sampling approach [12]. SECOAS (Self-organizing Collegiate Sensor Networks) considers a kind of smart sensor, which enable automated adaptation to upgrades and changes in network. It also tries to develop new technologies in a realistic application context [13].

To get the observed data efficiently, environmental monitoring applications require an effective data acquisition strategy. It needs to adapt to the application’s goals and functions. For example, local data summarization in pre-defined zones [14,15] exist; other strategies are all sensor data acquisition [15–17], intermediate data summarization [14], and an adaptive system as a self-organizing system [18]. The applications, which use the intermediate data summarization can identify zones of interest such as hot and cold zones such as maps of temperature and relative humidity [14]. To avoid information overload, they get the summarized data over subregions, pre-defined zones by spatial aggregation. For example, it uses an aggregation predicate such as “SELECT avg (volume) FROM Sensors GROUP BY region HAVING avg (volume) > threshold.” [14]. The spatial distribution of these summaries provides a report of the data variability over the entire region. An adaptive system for biology is described as a self-organizing system [18]. To understand the animals’ lives in ecosystem, we need to track their location and social interactions. This system uses an adaptive detection algorithm changed by the detected animal sounds. The goal of these tools is to perform autonomous wildlife monitoring as a powerful data collection system with pre-specified conditions.

## 3. Air Pollution Monitoring System

In this paper, we describe an air pollution monitoring system to analyze the sensor data with a context model and to adjust the sampling interval of sensors in a user defined area. To detect the conditions of a remote place, the monitoring system utilizes the measured data transmitted from a geosensor network. The geosensor network is a kind of sensor network which is designed to collect geospatial data [19,20]. To check the overall conditions, it basically uses a simple data transmission method, which sends sensor data to the network control system per every predefined sampling interval.

**Figure 1.**
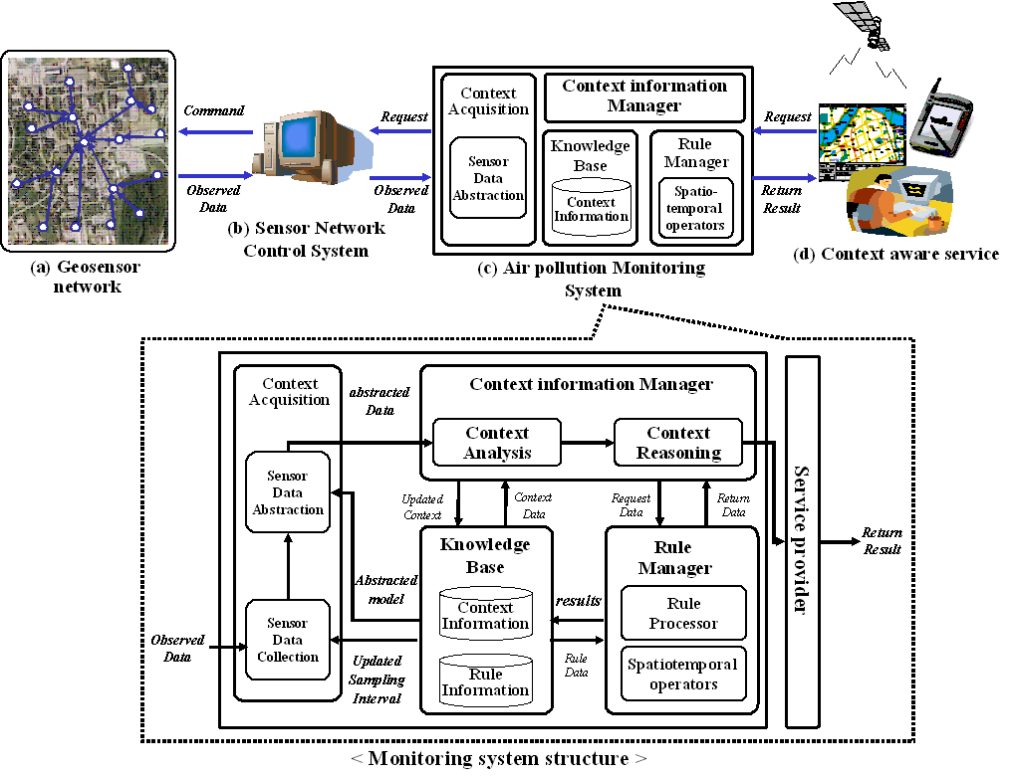
The architecture of context awareness system.

As shown in Figure 1, the system architecture consists of four parts; (a) geosensor network, (b) sensor network control server, (c) air pollution monitoring system, and (d) context-aware service. The geosensor network (a), transmits the measured data to the network control server per the defined sampling interval. The control server (b) manages the sensor network by using the control operators such as sampling interval change and network status check. The server collects all sensor data and provides the various kinds of monitoring systems simultaneously to the user and applications. The air pollution monitoring system, (c), is one of the monitoring systems, which receive the sensor data sent from the control server. It analyzes the current and potential pollution area. Finally, the alarm message and the safety guideline for pollution area, (d), are provided for people in the areas until the dangerous factors are gone.

**Figure 2.**
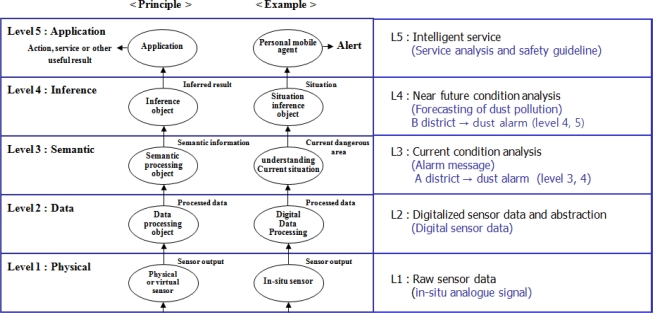
Sensor data abstraction steps.

Figure 2 shows the data processing steps in the air pollution monitoring service which is based on the abstraction [21]. In layer 1, it transmits their measured signals of the physical sensors which are installed in real world. It converts the received signals to the corresponding digitalized values to interpret the conditions in layer 2. In layer 3, it extracts the meaningful data and makes the useful information to understand the current situation of a remote place by using the context model with the user defined rules. It also predicts other information from the current situation depending on the applications in the inference step of layer 4. Data mining and other learning techniques can be used for this inference step. In layer 5, it provides application services depending on the analyzed situation. In this paper, we focus on the data analysis with context model in layer 3 to understand the conditions and the flexible sampling interval change to get data effectively.

**Figure 3.**
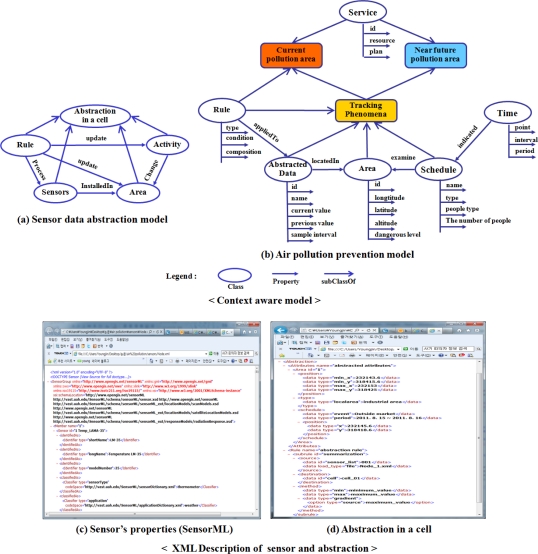
The context model for pollution prevention.

In order to detect the status of air pollution, Figure 3 shows the designed context models [4]; sensor data abstraction model for local data representation and air pollution prevention model for tracking pollution areas.

The main attribute of these models is a geographic region, because all activities of phenomena are based on geographic information such as the spread of air pollution. When the system processes the detected sensor data, these models are utilized to spatiotemporally analyze the pollution and to generate useful guidelines for prevention. To apply this model to the system, the classes of the model are described in xml format. For example, the monitoring system understands the attributes of the abstracted data such as position and size after loading the xml file. The rule of abstraction is also registered in the system as a function. When the system receives sensor data, the registered abstraction function for rule derives the abstraction in a cell by calculating the attributes of the abstraction such as min() and max() from sensor data.

## 4. Data Analysis for Pollution Prevention

**Figure 4.**
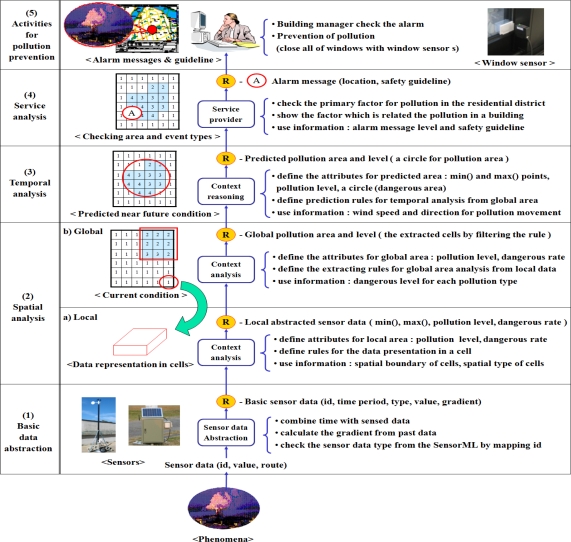
The data analysis steps for recognizing air pollution area.

To provide useful prevention information such as the status of pollution, safety guidelines, it should extract the meaningful data and understand the current and potential condition through spatial, temporal, and service analysis. Figure 4 shows the data analysis in the air pollution monitoring system.

It consists of five steps, from: (1) basic data abstraction to (5) activities for pollution prevention. For every step, we have to define the attributes of the results as data processing parameters of the next step. It also requires the definition of rules to make the values of attributes with the predefined information such as the properties of sensors, pollution type and level, cell size of grid, and the relation between pollution and resident district.

In this paper, the air pollution level follows the CAI (Comprehensive Air-quality Index) of the Ministry of Environment of South Korea to describe the ambient air quality based on the air pollution health risk [22]. In CAI, the pollution is measured per hour and per day. However, in order to provide an emergency alarm, our monitoring system measures air pollution in nearly real time by using a sensor network.

### 4.1. Basic Data Abstraction

The received sensor data in monitoring system is described by Definition 1. Id is a sensor id to check the observation type and its value. The route shows the data transmission path that is the list of sensors’ id or routers’ id which data passed. It is useful to check the connection among sensors. The collection module combines the received data with the properties of sensors in the sensorML [23]. The result of this layer is a basic sensor data form, which includes time period and gradient.

### 4.2. Spatial Analysis

In the spatial analysis layer, we use a grid structure which is utilized as a terrain surface model and a data representation structure in GIS [24]. A grid is an effective data structure to rapidly access spatial data by using a hash table [25]. When it handles large volume of data, it can promptly update and search the data using the hash function. In order to utilize the sensor data in a monitoring system, sensor data abstraction is required to simplify the data processing steps. For example, if it checks all of the sensor data to find the pollution area, it can require a long time to compute useful information depending on the number of sensors [26]. This step is divided into two parts; local and global area analysis. Global area presented by a grid, which is consists of a set of cells. A cell is represented by summarizing sensor data in the cell. Current pollution area is a subset of G, which has current high pollution value and near future pollution area is presented by a subset of G, which has the predicted high pollution value, which is derived by Algorithm 1.

***Definition 1.*** *(Sensors)* Let *S = { s_1_, s_2_, …,s_i_,…,s_n_ }* be a set of sensors in a two-dimensional Euclidean plane. Sensor *s* is a tuple of *(sensorid, sensortype, x, y, value, route)*.
***Definition 2.*** *(Cell)* Each cell of a grid is a tuple of *(cellid, min.x, min.y, min(), max.x, max.y, max(),gradient of max(), set of S). C = {s_1_, s_2_, … ,s_n_}*.
***Definition 3.*** *(Grid)* A grid G is a set of non-overlapped cells, in other word *G = {c_1_, c_2_,…, c_m_}*. A grid is a tuple of *(min.x, min.y, min(), max.x, max.y, max(),and gradient of max(), set of C).*

#### 

##### (a) Local area analysis

The local abstraction area shows data representation in a cell of a grid for presenting the part of pollution area. The value of each cell represents the pollution level in a cell in Figure 4. The cell size is defined by the number of sensors which is included in a cell, because it focuses on the sensor data representation such as min(), max(), and gradient. Max() and Min() shows the maximum and the minimum value of the detected sensor data in a cell. A gradient indicates the difference between past and current maximum values. This gradient is used to derive the probability of potential pollution of each cell. If two sensors are included in a cell, it is enough to make the local abstraction as shown in [26]. Besides, the system also calculates the dangerous rate, which indicates the probability to reach the critical point for dangerous pollution in the same way of Algorithm 1.

**Algorithm 1.** Global air pollution prediction with Gaussian air spread plume.

Algorithm predict_air_pollution (class pollution_area *current_pollution_area, class wind_info *wind)
**input:** current_pollution_area // the properties of global pollution area such as max() and min().
wind // the properties of a wind such as direction, speed.
**output:** predicted pollution level // the predicted value in 10, 30, 60 minutes
**method:**
// check the progress direction and get predicted pollution level
**for** each time // 10, 30 60 minutes
// get the moving position in each time
distance = wind.speed * time
**for** each position of current pollution area such as max(), min(), boundary
target.x = current_pollution_area.position.x + distance * cos(wind.direction * pi / 180)
target.y = current_pollution_area.position.y + distance * sin(wind.direction * pi / 180)
target.value = current_pollution_area.position.value
// pollution value prediction at each position in each time
pollution_level[time][position] = **Gaussian_air_pollution_dispersion**(time, current_pollution_area, target)
dangerous_rate[time][position]= pollution_level[time][position] / AQI(level_5) * 100 * gradient
**endfor**
**endfor**
**return** pollution_level[time][position], dangerous_rate[time][position]
**end**

##### (b) Global area analysis

The global abstraction area describes the overall pollution area, which is set from local abstraction areas by filtering rules. The local area is used to show a part of the pollution area. To make a global area by assembling these local areas, it employs user defined rules to extract specific area such as “dangerous rates of cells > 25%”, or “max() – min() in cells = 0.” The set of the extracted cells became a global abstraction area. In this paper, the system checks the local dangerous rate in cells over 15% and makes a global pollution area. The extracted area is used to understand which area is safe or dangerous. The system gives the alarm message and safety guideline to current polluted area.

**Figure 5.**
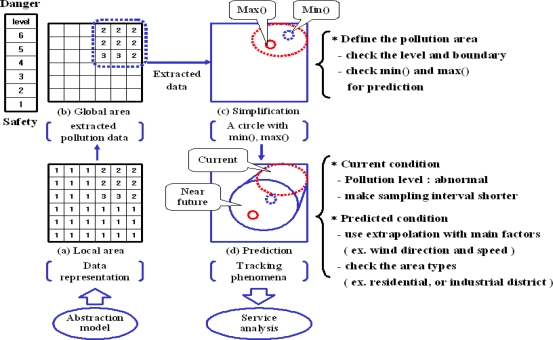
Sensor data processing for defining pollution area.

Figure 5 shows an example of the sensor data processing steps to define a potential dust pollution area. Dust sensors detect air pollution in the north and east parts of the map. The current dust level is 2∼3. It is not dangerous, but it could get worse. The system guesses that it could be an indication of air pollution in the near future and shortens the sampling interval of sensors in the current and the potential dust pollution areas to get more detailed data.

### 4.3. Temporal Analysis

It is useful to predict near future pollution levels and areas based on the current situation. The context reasoning module predicts the near future pollution areas based on the current pollution area(s) with user defined rules. It makes a circle to simply handle the current pollution area after getting the boundary of the global pollution area as shown in Figure 5. The circle includes two spots for min() and max() in pollution. As shown in Algorithm 1, the system employs a time-parameterized function with a trigonometric function to calculate the progress direction of the detected air pollution from wind direction and speed. With this derived direction, a Gaussian air pollution dispersion plume [27] is used to calculate current and near future pollution levels. To improve the accuracy of the determination of potential polluted areas and levels, it can use other good methods such as a data mining, or a statistical method. Whenever the global map is updated, the near future pollution area is also updated promptly.

### 4.4. Service Analysis

The service provider finds the overlap between the pollution area and a residential district and industrial area. For example, if a residential district is overlapped with a current or predicted pollution areas, the system checks the primary factor to estimate damage to the area and puts out an alarm message and safety guidelines. If the pollution areas are overlapped with a field which contains no people, it defines the area to be an off-limits zone depending on the pollution level. Besides, this system also provides the network status and controls the sampling interval to effectively collect data. To provide this service, it needs to define the area types, event types, and schedules with the related pollution type and level.

### 4.5. Activities for Pollution Prevention

This monitoring system shows the current and the potential pollution area for a system manager to understand the current situation and to protect from the pollution. For example, our system sends an alarm to a building in a potential polluted area. The alarm shows the critical factor that is the opened window, because the building air can be polluted from the detected outside air through the window. The alarm message turns off after closing the window, because there is no critical factor to pollute the air inside the building. This rapid prediction alarm is very important to reduce the potential damages and the recovery costs. Besides, this system provides network control operations for checking network status and for getting sensor data effectively such as the flexible sampling intervals.

## 5. Implementation

In order to check the environmental condition, we installed 10 routers and 24 sensors (Table 1) on a testbed field. It included various 12 types of sensors such as temperature, humidity, illumination, dust, carbon dioxide, ultraviolet radiation, wind direction, wind speed, air pressure, and altitude. By processing the real sensor data with context model, the air pollution monitoring system shows the implementation results.

**Figure 6.**
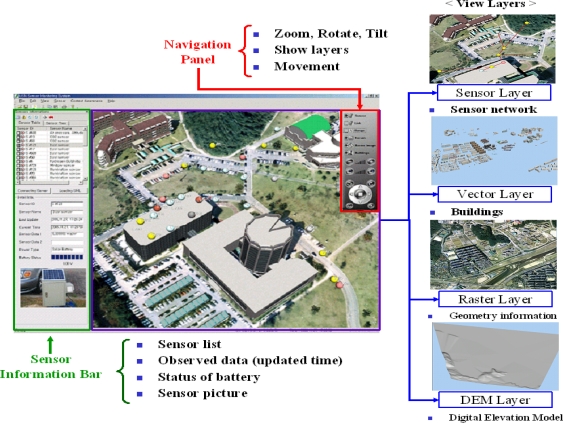
User interface for environmental monitoring.

Figure 6 shows the user interface of the air pollution monitoring system, which is based on the proposed framework for context awareness discussed in [28]. It consists of a sensor information bar, a view, and a navigation panel. The information bar presents the list and the properties for the registered sensors. When users click on a sensor in the list, it shows sensor ids, types, latest data, update time, the status of battery, and a stored picture of a sensor to check its type. This information is used for checking the current status of sensors. For example, if the update time indicates an hour before, it means that the sensor did not send the measured data from that time. It also checks the accuracy, the sensing range, and the sensing limit of sensors using the sensorML [23]. The view shows the geometry and sensor information in this application. To create a virtual reality view, four types of geometry data such as DEM (Digital Elevation Model), raster, vector, and geosensor layers are utilized. This extra data is used to provide foundation information such as a nearest hospital and police office. The navigation panel is used to control the view with some operators such as zoom in/out, rotation, tilt, movement, and view of layers.

**Figure 7.**
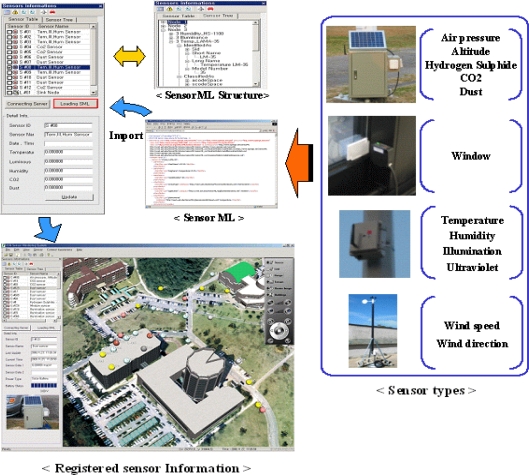
Sensor information management with sensorML.

In order to collect the information from the installed sensors, the proposed monitoring system uses sensorML, which describes the properties of sensors such as identification, constraints, coordinates, measurements, and documents in a standard XML schema. After loading the sensorML, the system can recognize the properties of the sensors such as id, location, type, accuracy, as shown in Figure 7. The sensors are presented in the view as colored spheres such as red (dust), yellow (illumination, temperature, humidity), blue (CO_2_, gateway, router), gray (hydrogen sulphide, ultraviolet radiation), black (air pressure, altitude), brown (window, wind direction, speed).

**Figure 8.**
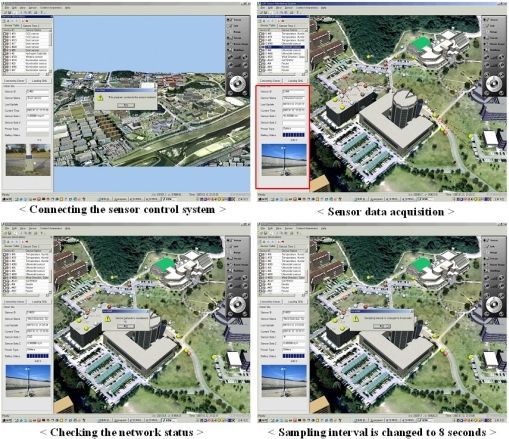
The sensor data acquisition, transmission routes, and the network control.

Figure 8 shows the implementation results to request sensor data, check the status of the sensor network, and adjust the sampling interval. The monitoring system connects the sensor network control server to get the sensor data. It provides the measured data to the connected monitoring system per every sampling interval. When the monitoring system receives the transmitted data, it shows the real time data transmission routes for checking the communication status in sensor network. The control server also manages the sensor network by adjusting the sampling interval, checking the network status. Besides, to get sensor data effectively, the sampling interval is automatically changed by the derived air pollution rate. The manager of the system also adjusts the sampling interval of sensors to check the current condition by using a scenario based sampling interval adjustment method [29].

**Figure 9.**
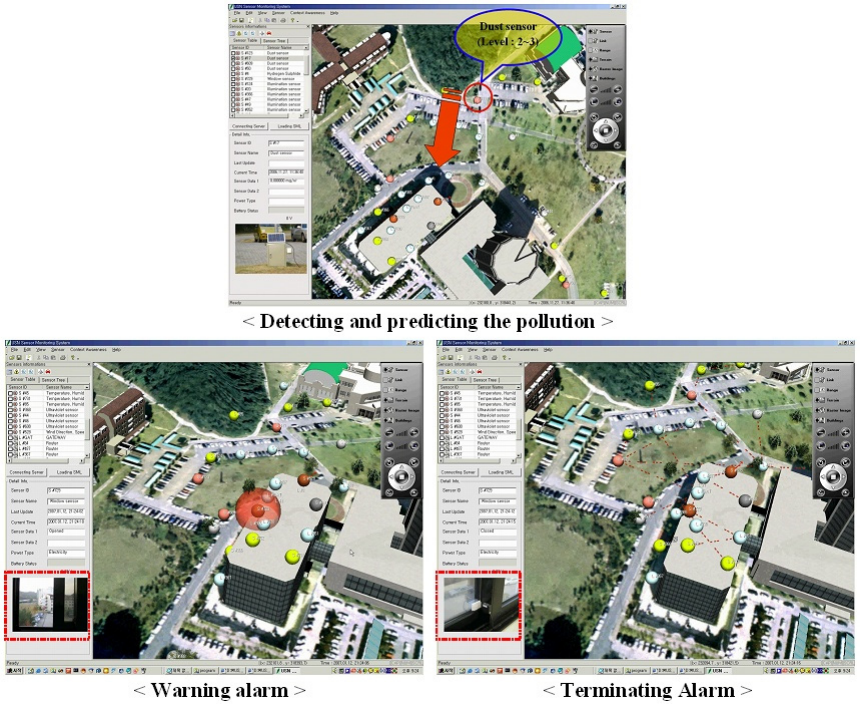
The provided alarm message and the safety guidelines.

Figure 9 shows the designed air pollution simulation scenario. When the observed data of a dust sensor is higher than the dangerous level with regard to the air pollution, the data analysis checks the current pollution area the cells around the sensor. It also checks the area types such as a school, a factory, and a forest, because the danger levels vary depending on the area type. After defining the current pollution area, it computes and predicts the potential pollution area in the near future using the related factors such as the pollution level gradient, the area type, wind direction and speed. When it finds a factor that predicts a dangerous condition in the near future, it creates an alarm message and displays it that until the factor is gone. The alarm message includes the pollution level and type, and safety guidelines.

To test the capability of danger recognition of the proposed data analysis, we used the simulated dust sensor data, because we cannot create real pollution. After updating the dust level, the system recognizes the pollution area and indicates a factor for the potential dangerous factor like the (a) of Figure 9. It shows an opened window of a building in a potential pollution area, because it is a primary factor for air pollution inside the building. The status of the window is also observed by a window condition detection sensor. The people in the building can recognize what the problem is and its factor. After closing the window, the alarm is terminated. It is very important to provide the predicted alarm message so we can take precautionary measures against the pollution. This proper estimation method of a pollution episode will utilize not only the detected sensor data but also weather condition. This system performance will be evaluated and improved by utilizing some air pollution study such as [30–35].

## 8. Conclusions

The designed air pollution monitoring system is useful for understanding current and near future pollution areas by utilizing a proposed sensor data analysis. This model employs an adaptive, flexible sampling interval changed by the status of the recognized situation. The economy in use and preserving electric power is really important in the sensor network because of limited battery power. As a next step, we are focusing on heterogeneous sensor data abstraction and the sensor data fusion for making a higher context in sensor network applications.

## Figures and Tables

**Table 1.** The installed sensor types.

Node types	Quantity	Node types	Quantity	Node types	Quantity
Gateway	1	Carbon dioxide	2	Dust	4
Router	10	Ultra violet	4	Wind speed, direction	1
Window	1	Illumination	6	Humidity	4
Hydrogen Sulfide	1	Air pressure/altitude	1	Temperature	
